# Workflow for predictive risk assessments of UVCBs: cheminformatics library design, QSAR, and read-across approaches applied to complex mixtures of metal naphthenates

**DOI:** 10.3389/ftox.2024.1452838

**Published:** 2024-10-01

**Authors:** A. J. Prussia, C. Welsh, T. S. Somers, P. Ruiz

**Affiliations:** ^1^ Office of Innovation and Analytics, Agency for Toxic Substances and Disease Registry, Atlanta, GA, United States; ^2^ Office of Community Health and Hazard Assessment, Agency for Toxic Substances and Disease Registry, Atlanta, GA, United States

**Keywords:** unknown or variable composition complex reaction products and biological materials (UVCBs), metal naphthenates, quantitative structure–activity relationships, quantitative structure–property relationships, read-across, virtual chemical library, cheminformatics, risk assessment

## Abstract

Substances of unknown or variable composition, complex reaction products, and biological materials (UVCBs) are commonly found in the environment. However, assessing their human toxicological risk is challenging due to their variable composition and many constituents. Metal naphthenate salts are one such category of UVCBs that are the reaction products of naphthenic acids with metals to form complex mixtures. Metal naphthenates are often found or used in household and industrial materials with potential for human exposure, but very few of these materials have been evaluated for causing human health hazards. Herein, we evaluate metal naphthenates using predictions derived from read-across and quantitative structure–activity/property relationship (QSAR/QSPR) models. Accordingly, we first built a computational chemistry library by enumerating the structures of naphthenic acids and derived 11,850 QSAR-acceptable structures; then, we used open and commercial *in silico* tools on these structures to predict a set of physicochemical properties and toxicity endpoints. We then compared the QSAR/QSPR predictions with available experimental data on naphthenic acids to provide a more complete picture of the contributions of the components to the toxicity profiles of metal naphthenate mixtures. The available systematic acute oral toxicity values (LD_50_) and QSAR LD_50_ predictions of all the naphthenic acid components indicated low concern for toxicity. The point of departure predictions for chronic repeated dose toxicity for the naphthenic acid components using QSAR models developed from studies on rats ranged from 25 to 50 mg/kg/day. These values are in good agreement with findings from studies on copper and zinc naphthenates, which had no observed adverse effect levels of 30 and 118 mg/kg/day, respectively. Hence, this study demonstrates how published *in silico* approaches can be used to identify the potential components of metal naphthenates for further testing, inform groupings of UVCBs such as naphthenates, as well as fill the data gaps using read-across and QSAR models to inform risk assessment.

## 1 Introduction

Consumers encounter various substances in their daily lives; some of these are pure chemicals like sugar, while some others are simple solutions of two chemicals, such as isopropyl alcohol dissolved in water. Other substances are more complex mixtures but still well-defined, such as ibuprofen formulated in a quick-release capsule. However, most of the substances exposed to consumers are highly complex mixtures, such as unknown or variable composition, complex reaction products, and biologicals materials (UVCBs) (Lai et al., 2022; Salvito et al., 2020); these complex substances often end up in the environment and may affect people. The Agency for Toxic Substances and Disease Registry (ATSDR) has been mandated with evaluating human exposures to many such substances (SARA, 1986). For individual chemicals or specifically defined mixtures, the ATSDR accomplishes its task by publishing the toxicological profiles of well-researched substances or by estimating the toxicity using computational methods (Sudweeks et al., 2024). Although exposures to UVCBs are common, their assessment as a substance class is challenging compared to the well-established protocols for evaluating single chemical compounds (Lai et al., 2022). UVCBs constitute a broad range of substances from natural-origin (e.g., petroleum fractions, essential oils) to synthetic (e.g., chlorinated paraffins, copolymers) products. Although some UVCBs contain structurally similar chemicals, others may contain diverse components with compositions that vary from batch to batch owing to the starting materials or reaction conditions and processes used. The three main approaches used for risk assessments of UVCBs are as follows: 1) evaluation of the whole substance, 2) evaluation of known constituents, and 3) fractional profiling. The use of these approaches depends on the exact nature of the UVCB; most UVCBs are assessed as whole substances, even though their potentially variable constituent solubilities could present challenging test conditions. Hence, relatively few UVCBs have been assessed for health risks, leaving a large gap in our toxicological knowledge of exposures. Filling this knowledge gap through animal testing of whole substances, known constituents, or fractional profiling entails ethical concerns. The results may also not be applicable for given variable UVCB compositions between preparations. There are several new approach methodologies suitable for single substances (e.g., *in vitro* assays, genomic expression analyses, computational models, read-across), but their application to UVCBs remains challenging because thousands of chemicals without known structural information could contribute to the effects of a UVCB (House et al., 2021). The European Chemicals Agency (ECHA) developed guidelines for the grouping and read-across of UVCBs to meet the requirements of the REACH regulation (European Chemicals Agency, 2017); however, its application is currently limited to UVCBs with minor differences in the constituents. Moreover, the Organization of Economic and Co-operative Development (OECD) grouping guidance remains limited for UVCBs, although this may change since it is being revised (OECD, 2017). In short, the currently available *in vivo*, *in vitro*, and *in silico* tools for assessing UVCBs are inadequate.

UVCBs may be described using a hierarchy of structural characterizations from (a) well-defined individual structures, to (b) descriptive representative structures, (c) recognized external identifiers with some standardized descriptions, and (d) simple text-based descriptions (Lai et al., 2022). The most granular characterization is the best for assessing a UVCB, but this level of representation is not possible for many UVCBs that have thousands of inseparable constituents. Many UVCBs have descriptive representative structures with a basic scaffold and known structural units that result in variations or attachments to the scaffold. These UVCBs present opportunities for the application of cheminformatics approaches by enumeration of all possible structures to permit structure-based predictions or read-across. The last two structural characterizations are challenging for cheminformatics, but advances in analytical chemistry techniques such as high-resolution mass spectrometry have enabled describing these UVCBs structurally in conjunction with expert judgment. A recent example of this was for bromo-chloro alkenes, which are UVCB flame retardants (Chibwe et al., 2019).

**FIGURE 1:**
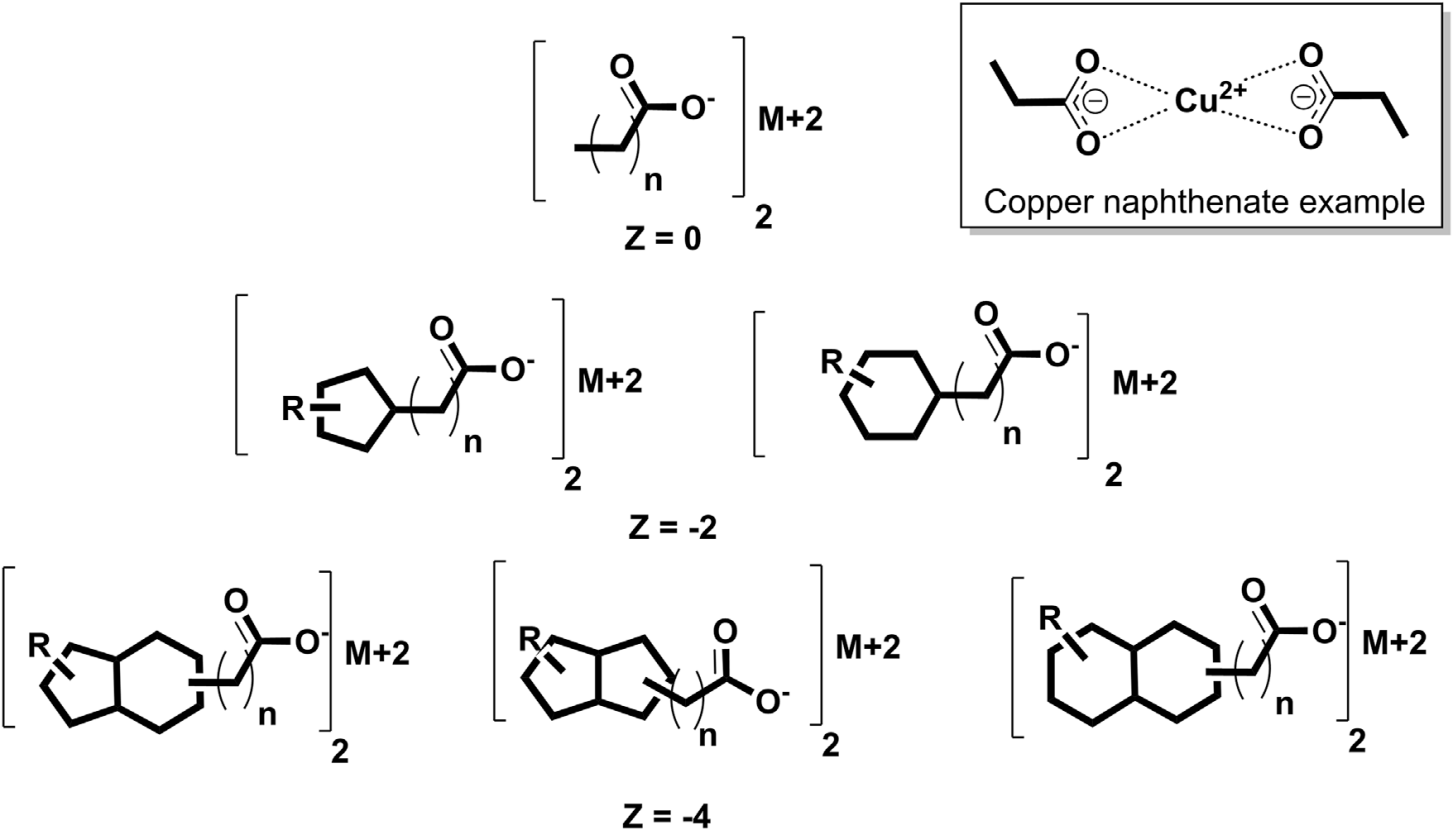
Sample naphthenic acid structures, where M+2 is the metal cation, R is an alkyl chain, Z is the hydrogen deficiency, and n is the number of CH_2_ units. A copper naphthenate example structure is shown in the top-right box. More negative Z structures, such as tricyclic compounds, are described in the supporting information.

Emergency situations involving UVCB exposures with limited toxicological information are especially problematic. Typical approaches involving read-across or quantitative structure–activity relationships (QSARs) that can rapidly help determine health guidance values (HGVs) for pure chemicals or simple mixtures are not readily applicable to UVCBs (Dimitrov et al., 2015; Kutsarova et al., 2019). Estimating the general range of physical properties of a UVCB may also prove difficult in such instances. These issues are apparent in the ATSDR through exposure incidents involving UVCBs over the past several years. In one incident, the Vermont Department of Agriculture’s state veterinarian sought the ATSDR’s technical assistance regarding exposure of dairy goats to copper naphthenate (CAS 1338-02-9), which is one of the possible metal naphthenates (Figure 1) (https://www.atsdr.cdc.gov/2021-annual-report/listening-responding-taking-action/Simulation-Science.html). As a fungicide with copper as the active ingredient and naphthenates as the carriers, copper naphthenate is associated with a regulatory assessment made by the US Environmental Protection Agency (USEPA) under the Federal Insecticide, Fungicide, and Rodenticide Act (FIFRA); however, its dietary risks were beyond the scope of the assessment since there were no registered direct or indirect food uses, with negligible expected exposure through drinking water (USEPA, 2018). Thus, several gaps remain in the evaluation of metal naphthenate exposure to the general public, as demonstrated by this incident. First, copper naphthenate has been examined as a single complete substance based on a toxicity study in rats. As a UVCB, the composition of a naphthenate varies depending on the source and refining methods used (Brient et al., 2000). If other preparations have toxic components constituting a greater percentage of the substance, their risk assessments will be underpredicted. Second, the oral and dermal exposure risks to the general population may be more common than expected, given the popularity of readily available do-it-yourself home improvement products. Third, there could be health risks to the general population from other metal naphthenates; although zinc naphthenate was assessed by the USEPA in 2007, no other metal naphthenates have been subjected to authoritative risk assessments by a regulatory agency in so far as we have been able to determine.

Given the number of metal naphthenates available in the market and limited resources for experimental testing, traditional toxicological assessment methods recommended for mixtures are inadequate (ATSDR, 2018). Multipronged approaches using established *in silico* tools could be alternatives to filling these data gaps through predictions of the toxicological properties based on computational models. These tools and models have advanced significantly over the past two decades, incorporating advanced computing capabilities, toxicological databases, and machine learning approaches. The purpose of this study was to address data gaps for metal naphthenates using a computational cheminformatic workflow through the following steps: i) developing a virtual chemical library of naphthenic acids; ii) performing read-across using analogs with experimental toxicological data; iii) applying QSAR models to predict the physical properties and mammalian toxicity values (i.e., acute oral toxicity, repeated dose and developmental toxicity endpoints) for the virtual library developed in (i); iv) calculating the estimates of metal exposure and subsequent risk assessments based on different metal naphthenate compositions by considering the contributions of the metal and organic naphthenate components separately. Through this workflow, we aim to enhance the capabilities of the ATSDR and other environmental health organizations to rapidly and effectively assess UVCBs for better preparedness with handling future situations involving UVCB exposures.

## 2 Methods

**FIGURE 2:**
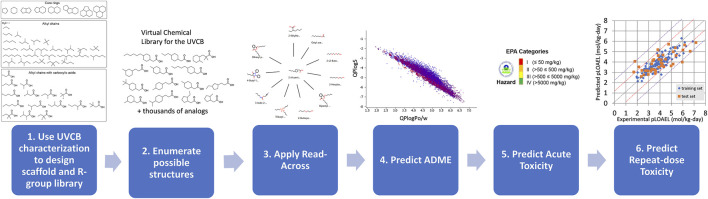
Overall methodology for predictive risk assessment of a UVCB. Step 1: The scaffolds and R-group library are designed based on UVCB characterization. Step 2: Cheminformatics tools are used to enumerate possible structures. Steps 3, 4, 5, and 6: Further *in silico* technologies (read-across, ADME, LD_50_, and repeat-dose toxicity predictions) are applied for the thousands of component structures.

Herein, we propose a methodology for the predictive risk assessments of UVCBs as applied to metal naphthenates. We constructed an integrated approach using cheminformatics, established read-across methods, and validated *in silico* models (Figure 2). First, we use the characterizations of the organic components in the metal naphthenates to design scaffolds and R-groups. Next, a structure library is created by enumerating the scaffolds with the R-groups. Finally, this allowed us to apply read-across tools and QSAR-based model predictions to determine the physicochemical, biological, and toxicological properties. All of the software tools, databases, and QSAR models used in the proposed method are shown in Table 1, and open-source alternatives are highlighted where applicable.

**TABLE 1:** Cheminformatics software, tools, and models used to predict UVCBs in this study.

Cheminformatics software tool, database, or model	Commercial or open-source product	Purpose	Open-source alternative	Selected result	Results in the virtual naphthenate library
Maestro enumeration tool	Commercial (Schrödinger, LLC)	Generation of the virtual chemical library of naphthenates	RDKit’s enumeration method	NA	11,850 naphthenate structures generated
KNIME analysis platform	Open source	Generation of InChi keys and CDK descriptors	NA	NA	InChi keys and CDK descriptors for all generated structures
Canvas cheminformatics platform	Commercial (Schrödinger, LLC)	Chemical data management for the naphthenate library	KNIME can be used as a standalone tool to manage the cheminformatics data	NA	Visualization of all structures and graphing data
EPA CompTox Dashboard	Open source	Allows searching of the DSSTox database	NA	NA	Identified 14 naphthenic acids in DSSTox
Generalized read-across web application	Open source	Read-across predictions of the naphthenate library	NA	*In vivo* POD predictions	POD predictions for 14 naphthenic acids, ranging from 1.7 to 4,531 mg/kg/day
QikProp prediction models	Commercial (Schrödinger, LLC)	Prediction of the physicochemical and pharmacokinetic properties	OPERA 2.9 also predicts many of the same properties	Octanol/water partition (log)	1.9 to 6.8
				Water solubility (log mol/L)	−1.8 to −7.8
OPERA 2.9	Open source	Prediction of the physicochemical properties, environmental fates, and toxicological endpoints	NA	Unbound plasma fraction	89% of the structures in the library are predicted to have an unbound fraction <0.2
				Hepatic intrinsic clearance (µL/min/10^6^ cells)	5–30 μL/min/10^6^ cells for most compounds
				Rat oral acute LD_50_ (mg/kg)	2,000–9,011 mg/kg
Repeat-dose POD prediction (Pradeep et al., 2020)	Open source (uses Python, NumPy, Scikit-learn, CDK, and PaDEL)	Prediction of PODs for chronic, subchronic, reproductive, developmental, and subacute studies in rats, mice, and rabbits	NA	Rat developmental POD (mg/kg/day)	79 to 95
				Rat chronic POD (mg/kg/day)	25 to 50

### 2.1 Development of a virtual chemical library of naphthenic acids

**FIGURE 3:**
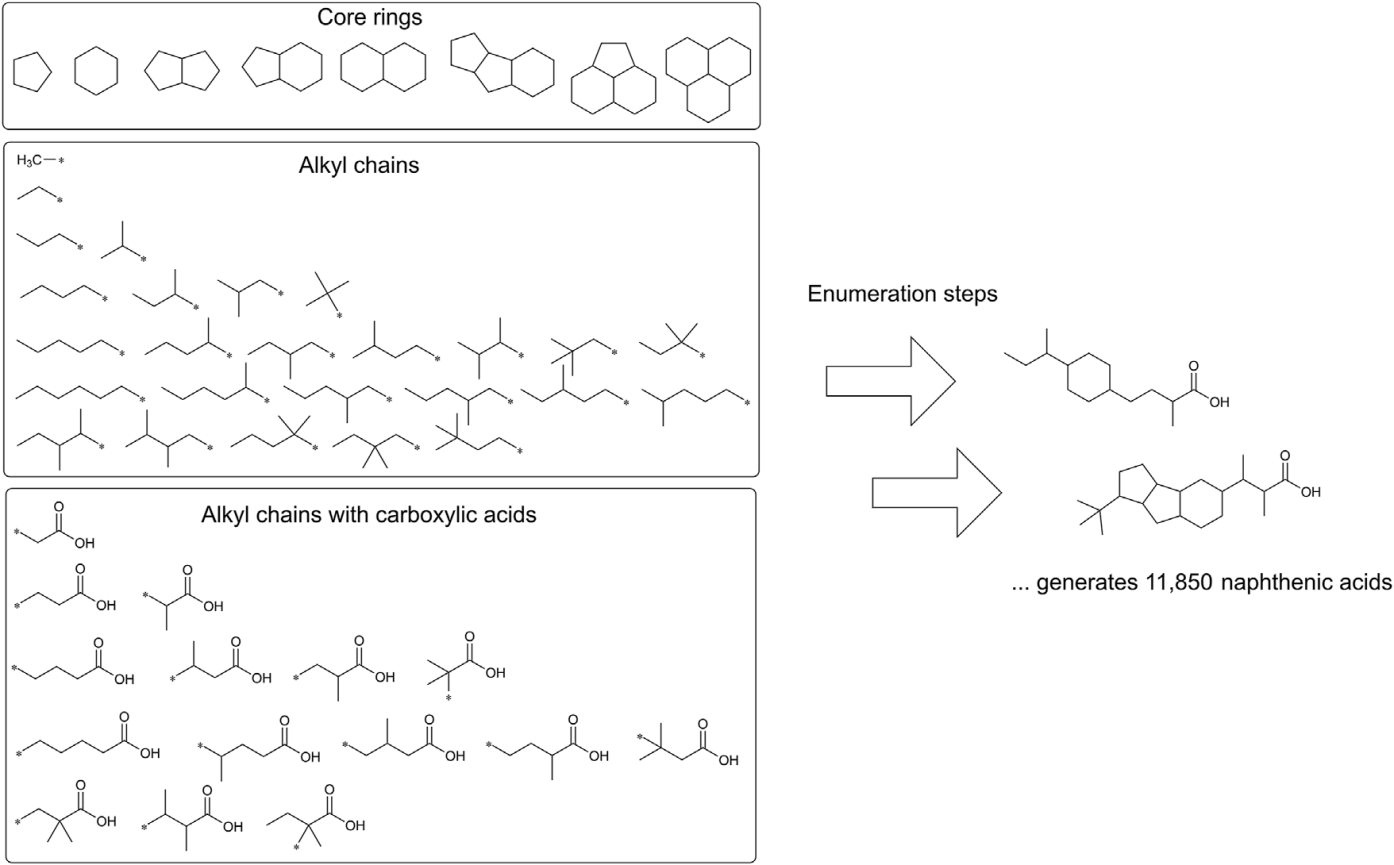
Creation of a virtual chemical library of naphthenic acids. Through two enumeration steps, 26 alkyl chains (middle) and 15 alkyl chains with carboxylic acids (bottom) were attached to the eight core rings. The attachment points are indicated by asterisks. The possible combinations of R-groups and ring positions resulted in the derivation of 11,850 unique naphthenic acid structures.

A virtual chemical library of the organic components of metal naphthenates was developed using the eight core ring structures observed in experimental samples of naphthenic acids and the commercial molecular modeling tool Maestro (version 12.5, https://newsite.schrodinger.com/platform/products/maestro/) (Figure 3). The enumeration tool was used to first define the attachment points (Schrödinger LLC, 2020b); the R-groups for the enumerations were built with 26 alkyl chains for methyl, ethyl, propyl, butyl, pentyl, hexyl, and their respective isomers with attachment points. Similarly, the R-groups for 15 alkyl chains with carboxylic acids were built with attachment points. Through these two enumeration steps, each core ring structure was attached to one alkyl chain R-group and one carboxylic acid alkyl chain R-group for all possible positional isomers. These core rings and R-groups were selected on the basis of the structural characterizations in Brient et al. (2000), and the resulting combinations were used to generate a library of QSAR-suitable structures (Supplementary Material S1). Using the open-source KNIME Analytics platform (version 4.7, www.knime.com) and available InChI-based nodes within KNIME, the library was translated from the native Maestro format to other suitable formats, such as InChI keys, for further cheminformatic analyses (Berthold et al., 2008).

### 2.2 Read-across with EPA CompTox Chemicals Dashboard and Generalized Read-Across (GenRA)

To identify whether any of the enumerated structures (i.e., organic components of the metal naphthenates) matched the registered substances in the Distributed Structure-Searchable Toxicity (DSSTox) database (Grulke et al., 2019) underpinning to the EPA CompTox Chemicals dashboard (version 2.1.2, https://comptox.epa.gov/dashboard), InChI keys were used in a batch search (Williams et al., 2017). Structures in the naphthenic acid library were exported from KNIME as InChIKeys and searched using the InChIKey skeletons (Berthold et al., 2008). Searching as a skeleton ignores the stereochemistry, bond types, charge states, tautomerism, and isotopes to maximize the number of potential hits. The chemicals matching the structures in DSSTox were identified, and their associated metadata (safety data, ToxCast assays results, etc.) were downloaded. The GenRA web application (Patlewicz and Shah, 2023) (https://comptox.epa.gov/genra/) was then used for these chemicals along with the Morgan chemical fingerprints and ToxRefDB v2 database (Helman et al., 2019; Watford et al., 2019). Between two and six read-across analogs were identified for each chemical depending on functional group similarities, and similarity-weighted read-across calculations were performed. Each endpoint with data for read-across analogs was captured in a spreadsheet.

### 2.3 QSAR predictions

QSAR models were applied to the virtual chemical library for physicochemical, pharmacokinetic, biological, environmental fate, and toxicity predictions, including acute oral toxicity and repeat-dose toxicity for the points of departure (PODs). Within the commercial cheminformatics tool Canvas 4.5 (Schrödinger LLC, 2020a), the physicochemical and pharmacokinetic properties were predicted using the suite of models available in Schrodinger’s QikProp 6.5 for the virtual chemical library, including solubility, octanol/water partitioning, and degree of cyclization (Schrödinger LLC, 2020c). The open-source tool OPERA 2.9 was used for batch prediction of all the available models, including predictions for environmental fate, transport, and acute oral toxicity (Kleinstreuer et al., 2018; Mansouri et al., 2018; Mansouri et al., 2021b). The absorption, distribution, metabolism, and elimination (ADME) predictions were also obtained using QikProp and OPERA 2.9. The repeat-dose toxicity PODs were predicted using the models developed by Pradeep et al. (2020). These QSAR models were chosen for their wide variety of pharmaceutically relevant property predictions (QikProp 6.5), established consensus models based on global collaborations (OPERA 2.9), or large training sets of *in vivo* toxicity data used to build the models (Pradeep et al., 2020). Other QSAR models could also be applied to yield insightful results.

### 2.4 Metal naphthenate exposure analyses and estimates

Based on the experimental molar acid count of a naphthenic acid mixture and its molecular weight range (Brient et al., 2000), the compositions were estimated for copper, zinc, and cobalt naphthenates. Based on these compositions, the estimates of the metal and concurrent naphthenate exposures were developed for these UVCBs, with the metals being considered in the +2 oxidation states. For these metal naphthenates, it was assumed that the salts dissociated in solution and could therefore be assessed as concurrent metal and organic exposures. The ATSDR HGVs, known as Minimal Risks Levels (MRLs), for the metals were then compared to the predicted PODs for the naphthenic acids, and health guidance suggestions were provided for the copper, zinc, and cobalt naphthenates.

## 3 Results

### 3.1 Development of a virtual chemical library of naphthenic acids

The virtual chemical library of naphthenic acids (i.e., organic components of metal naphthenates) was developed using the combinatorial chemistry tools available within Maestro 12.5. Similar computational facilities are also available from open-source tools, such as RDkit (Table 2); however, the graphical interface provided by Maestro simplified the construction of the core rings, alkyl chains, alkyl chains with carboxylic acids, and subsequent enumeration steps. According to the procedure described above, a total of 11,850 QSAR-suitable structures were determined (Figure 3). Building a virtual library in this manner necessarily encompasses some chemical entities that represent only a small molar fraction of the overall population of naphthenic acids. However, predictions are needed even for these less-common chemicals in the mixture since their toxicological properties may be markedly different.

**TABLE 2:** Metal naphthenates, composition ranges, predicted doses, and MRL-like values.

Metal naphthenate	Available experimental PODs	Metal naphthenate HGV equivalent^a^	Lower–upper bound %weight for metal^b^	Metal dose from metal naphthenates	ATSDR MRL for metal	Predicted driver of toxicology	Provisional recommendation
			Lower–upper bound %weight for naphthenates	Naphthenate dose from metal naphthenates			
Copper naphthenates	30 mg/kg/day rat developmental NOAEL (USEPA, 2018)	0.3 mg/kg/day	11.3–13.3	0.034–0.040 mg/kg/day	0.01 mg/kg/day (ATSDR, 2004a)	Copper	Based on copper exposure, limit copper naphthenate to 0.09 mg/kg/day
			86.7–88.7	0.26–0.27 mg/kg/day			
Zinc naphthenates	118 mg/kg/day rats developmental NOAEL (USEPA, 2007)	1.2 mg/kg/day	11.6–13.6	0.14–0.16 mg/kg/day	0.3 mg/kg/day (ATSDR, 2005)	Naphthenates	Based on naphthenates, limit zinc naphthenate exposure to 1.1 mg/kg/day
			86.4–88.4	1.04–1.06 mg/kg/day			
Cobalt naphthenates	–	–	10.6–12.4	For 1.2 mg/kg/day of metal naphthenates, 0.13–0.15mg/kg/day	0.01 mg/kg/day (ATSDR, 2004b)	Cobalt	Based on cobalt exposure, limit cobalt naphthenate exposure to 0.1 mg/kg/day
			87.6–89.4	For 1.2 mg/kg/day of metal naphthenates, 1.05–1.07 mg/kg/day			

^a^ Using the cited no observed adverse effect level (NOAEL) as the point of departure (POD) as well as applying uncertainty factors of 10 for animal to human extrapolation and 10 for human variability.

^b^ Percentage compositions based on KOH acid numbers given in Brient et al. (2000).

**FIGURE 4:**
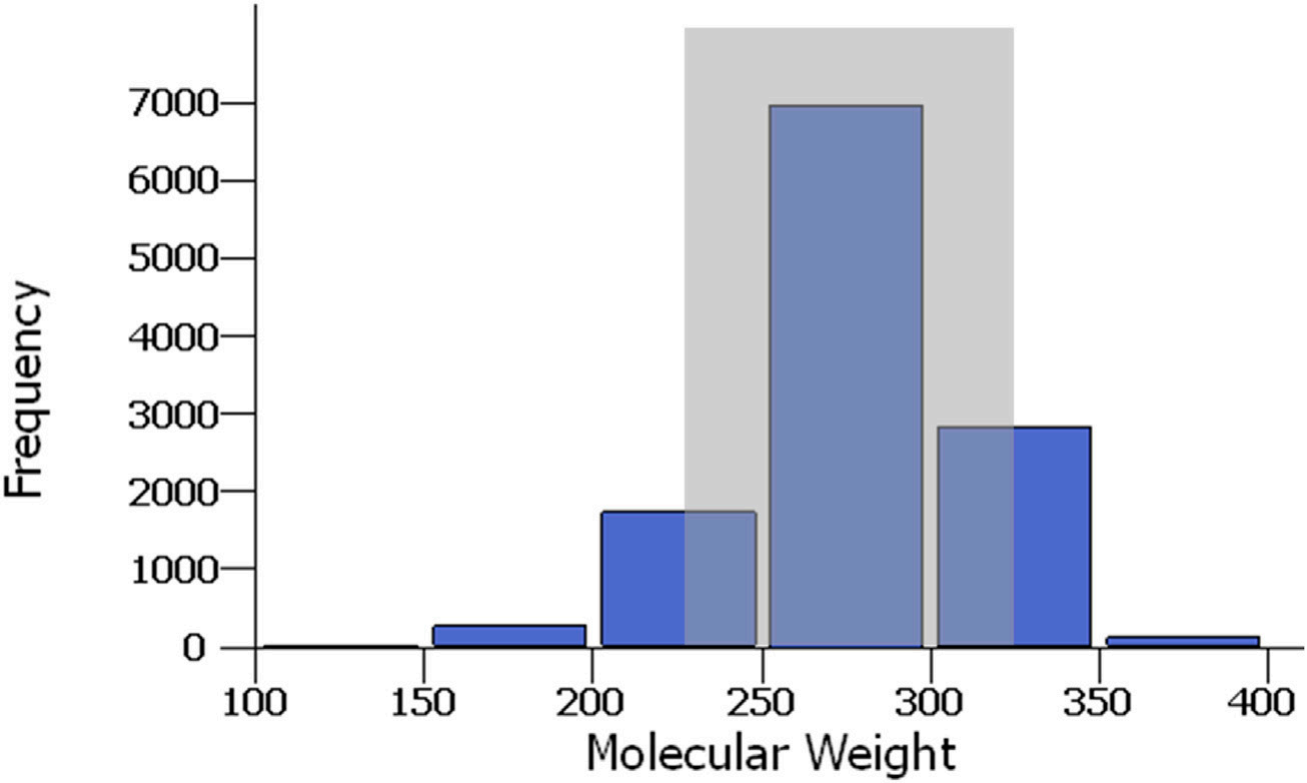
Distribution of molecular weights for the naphthenic acids in the virtual library. The gray box shows the experimentally derived range of mean molecular weights for crude naphthenic acids.

The overall physical characteristics of the naphthenic acids are matched well with those in the virtual library. For example, the average oil-free molecular weight for crude naphthenic acids is between 240 and 330 g/mol (Brient et al., 2000), and this corresponds well with the distribution of molecular weights in the virtual library, where most constituents are between 200 and 350 g/mol (Figure 4).

### 3.2 Read-across with the EPA CompTox dashboard

**FIGURE 5:**
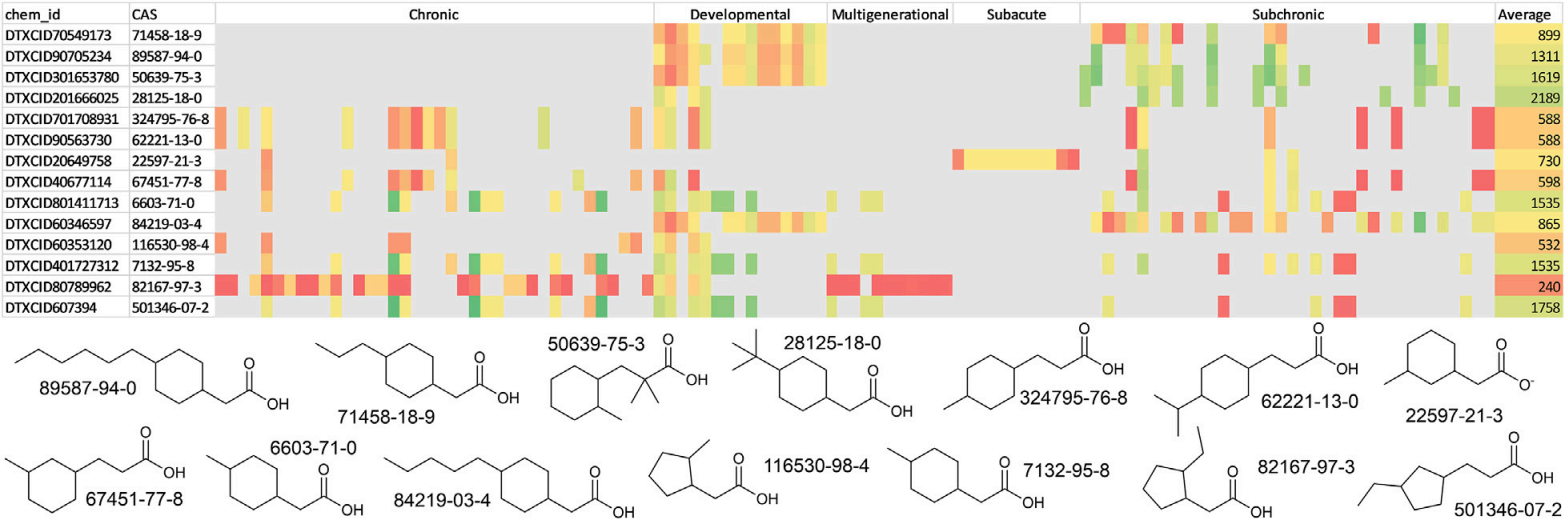
Generalized read-across predictions for the 14 naphthenic acids identified using the EPA CompTox Chemicals dashboard. The structures are shown along with their CAS numbers. The read-across prediction for each toxicity outcome is shown on a scale of red to green (red: 1.7–250; orange: 250–600; yellow: 600–1,200; green: 1,200–4,531 mg/kg/day).

All the structures from the naphthenic acid library were searched in the EPA CompTox dashboard (Williams et al., 2017). Of the 11,850 structures searched, only 14 unique substances were identified from among all the chemicals in the database, suggesting that the substances included in this specific chemical space lacked extensive documentation and experimental information in extant toxicological databases. In fact, the metadata associated with these compounds did not have any available bioactivity data or experimental physicochemical properties. The 14 compounds identified were then used with the GenRA web application to predict several *in vivo* toxicity outcomes (Figure 5) (Helman et al., 2019). The complete read-across results for each compound are included in Supplementary Material S2. To summarize, the read-across predictions for each of the toxicity outcomes were color coded from red (indicating the lowest doses for toxicities) to green (indicating high doses). The lowest value found was 1.7 mg/kg/day for chronic cardiotoxicity for the chemical DTXCID80789962 (CAS 82167-97-3). The least potent toxicity value was 4,531 mg/kg/day for chronic hematotoxicity for the chemical DTXCID607394 (CAS 501346-07-2). The mean POD values across all toxicity outcomes for the 14 chemicals ranged from 240 to 2,189 mg/kg/day.

### 3.3 QSAR predictions

#### 3.3.1 Physicochemical and ADME predictions

**FIGURE 6:**
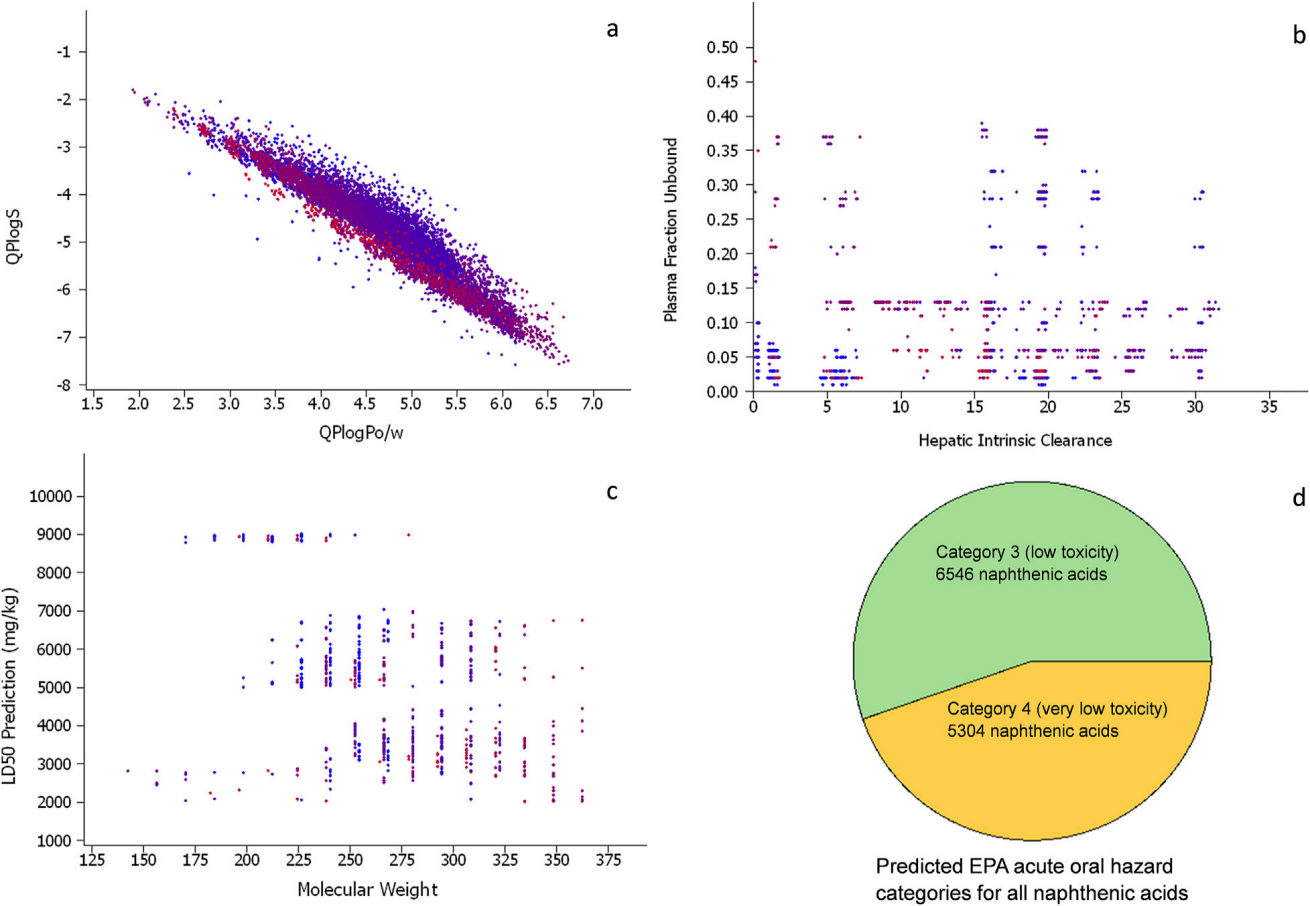
**(A)** Scatter plot of the predicted octanol/water partitions and water solubilities (in log units) for all naphthenic acids in the virtual chemical library (each point represents one chemical). **(B)** Scatter plot of predicted human hepatic intrinsic clearance (µL/min/10^6^ cells) *versus* predicted human unbound plasma fraction. All panels have the same numbers of points, and the molecules are colored according to cyclization degrees of the structures ranging from blue (low) to red (high). **(C)** Scatter plot of the predicted median lethal oral dose (LD_50_) in rats *versus* molecular weight for all naphthenic acids in the virtual chemical library. **(D)** Pie chart of the predicted EPA acute oral hazard categories for the naphthenic acids, with category 3 being “low toxicity” and category 4 being “very low toxicity.” No chemicals were predicted in the more toxic categories 1 and 2.

The naphthenic acids have wide ranges of values for the selected physicochemical property predictions, such as water solubility and octanol/water partitioning, which are consistent with their amphiphilic structures comprising an alkyl group attached to a carboxylic acid (Figure 6A). The cyclization degree of the molecules were calculated using QikProp (Schrödinger LLC, 2020c) and shown in the color range from blue (representing the least number of heavy atoms in a cyclic structure, 28%) to red (most cyclized structure with 72% heavy atoms). The cyclization degree does not appear to be highly correlated with solubility or partitioning, implying that cycloalkyls, branched alkyls, or linear alkyls can all function as hydrophobic tails in the molecules.

OPERA (v2.9) models for the ADME properties, such as hepatic intrinsic clearance and unbound fraction in plasma (Mansouri et al., 2018), were applied to the target chemicals (Figure 6B). Most of the structures in the library (∼89%) were predicted to have unbound fractions of less than 0.2; this is consistent with acidic compounds bound to albumin (Smith et al., 2019). The hepatic clearance was predicted to be between 5 and 30 μL/min/10^6^ cells for most compounds, which is near the median value for the 1,056 environmental chemicals used to develop the OPERA model (with values ranging from 0.01 to 10,000 μL/min/10^6^ cells) (Mansouri et al., 2021a; Watford et al., 2019). These predicted parameters suggest moderate to slow systemic clearances for most of the naphthenic acids. However, further modeling beyond the scope of this paper would be necessary to derive more complete toxicokinetic estimates.

#### 3.3.2 Acute oral toxicity predictions

The OPERA 2.9 model suite comprises recently added predictive tools for assessing systematic acute oral toxicity based on a global collaboration, yielding a consensus model with a performance equivalent to the variability of the experimental studies for median lethal dose (LD_50_) point estimates and an overall balanced accuracy of 0.8 for the EPA hazard categories (Kleinstreuer et al., 2018; Mansouri et al., 2021b). The LD_50_ point estimates in rats as well as classification models relying on the EPA or Globally Harmonized System of Classification and Labeling of Chemicals (GHS) hazard categories were applied (Figure 6C). All of the structures in the virtual library were predicted to have rat oral LD_50_ values between 2,000 and 9,011 mg/kg based on the point estimate model. Based on the classification models, all chemicals were found to be in EPA hazard category 3 (low toxicity) or category 4 (very low toxicity, Figure 6D). The GHS hazard category model predictions showed that all chemicals were in GHS category 5 (LD_50_ in the range of 2,000–5,000 mg/kg, i.e., “relatively low acute toxicity hazard”). Direct comparisons with the experimental oral LD_50_ values were not possible for any of the structures in the library owing to the lack of data. However, for the naphthenic acids as a whole UVCB substance, an experimental rat LD_50_ of 3,000 mg/kg was identified (Sigma-Aldrich, 2022); this is consistent with the predicted range of 2,000–9,000 mg/kg for all individual naphthenic acid components.

#### 3.3.3 Repeat-dose oral toxicity predictions

**FIGURE 7:**
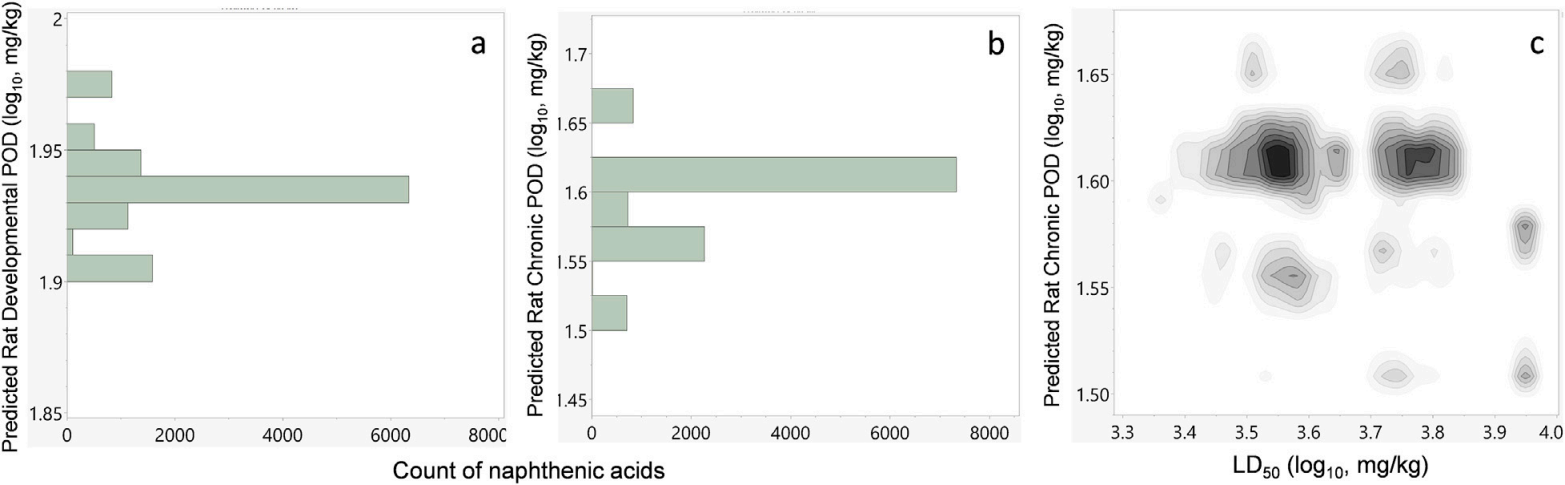
**(A)** Histogram of the predicted rat developmental POD values for all naphthenic acids in the virtual chemical library. The antilog-transformed values are equivalent to 79–95 mg/kg/day. **(B)** Histogram of the predicted rat chronic POD values. The antilog equivalents are between 25 and 50 mg/kg/day. **(C)** Density plot showing the naphthenic acid population with their predicted median lethal oral doses in rats (LD_50_) and predicted rat chronic POD values.

Repeat-dose oral toxicity is a challenging endpoint to predict but is the most relevant for human health risk assessment under environmental chemical exposure. Several models have been proposed to predict different effect levels, such as the lowest observed adverse effect level (LOAEL) (Mazzatorta et al., 2008; Mumtaz et al., 1995) and no observed adverse effect level (NOAEL) (Hisaki et al., 2015). This is significant because these types of effect levels provide the risk assessors with quantitative POD values that form the basis for extrapolation to HGVs such as the MRL (Chou et al., 1998; Chou et al., 2002) and reference dose (RfD) (Barnes et al., 1988). The Center for Computational Toxicology and Exposure at the USEPA has recently compiled an *in vivo* toxicity dataset of over 3,000 chemicals based on experimental repeat-dose toxicity studies in mammals (Watford et al., 2019). This rich dataset not only enables use of the GenRA web application but also provides a publicly available resource for developing predictive models. This dataset has also been used to develop models for predicting the PODs for chronic, subchronic, reproductive, developmental, and subacute studies in rat, mice, and rabbits (Pradeep et al., 2020). These models are publicly available and can be used with open-source tools, such as NumPy, Scikit-learn, PaDEL, and CDK. The PODs were also calculated for the proposed virtual naphthenic acid library using these models and open-source tools (Figures 7A, B).

The predicted repeat-dose chronic oral and developmental toxicity in rats is shown in Figure 7, while those for the other species and study types are available in Supplementary Material S3. The PODs are presented in units of mg/kg/day in log10-transformed values for easy display of data. For the rat developmental endpoint, all the naphthenic acids were predicted to have PODs between 1.90 and 1.98 log_10_-mg/kg/day (equivalent to 79–95 mg/kg/day, Figure 7A). For the rat chronic endpoint, most naphthenic acids had PODs between 1.4 and 1.7 log_10_-mg/kg/day (equivalent to 25–50 mg/kg/day, Figure 7B). As with acute toxicity, it is not possible to compare these results directly with individual experimental repeat-dose studies since such data do not exist. However, experiments have been conducted for naphthenic acids as a whole UVCB substance; Rogers et al. (2002) observed adverse effects of naphthenic acids at 60 mg/kg/day in rats dosed for 90 days. These results are consistent with the repeat-dose toxicity predictions for chronic rat PODs of between 25 and 50 mg/kg/day for all individual naphthenic acids.

### 3.4 Metal naphthenate composition and exposure estimates

To estimate the metal and organic fractions of copper, zinc, and cobalt naphthenates, their stoichiometries and reaction conditions were considered. Since naphthenic acids can be characterized by titration with potassium hydroxide to obtain the acid number for a particular composition, estimates of the metal compositions can be determined for the metal naphthenates. For example, refined naphthenic acids have acid numbers ranging from 225 to 270 mg KOH/g of naphthenic acid. This corresponds to 4.0–4.8 mmol KOH for neutralizing 1 g of the naphthenic acids assuming complete reaction conditions (Brient et al., 2000). Because the metal cation to naphthenic acid stoichiometry is 1:2 for metals in the +2 oxidation state, one-half the number of moles of metal (i.e., 2.0–2.4 mmol) would be needed to complex with the acids, assuming that the impurities are negligible and that the reaction is complete. Converting this molar quantity to mass based on the atomic weight of the metal results in metal naphthenates with percentage weights shown in Table 2. These compositions are confirmed in the elemental analyses of commercial products from Nisus and Alfa Aesar (Nisus Corporation, 2013; Alfa Aesar, 2023a; Alfa Aesar, 2023b). Because metal naphthenates dissociate into metal cations and organic anions in aqueous oral exposure, knowing the metal and naphthenate compositions allows separate risk assessments based on the ingested amount. For example, copper naphthenate can be treated as 11.3%–13.3% exposure to copper and 88.7%–86.7% exposure to naphthenic acids (both percentages by weight, Table 2). Note that this is under the assumption of complete dissociation of the metal and organic components.

Using the estimated compositions and results of available animal studies, it can be determined if the toxicology for a metal naphthenate is driven by the metal or naphthenate components. Developmental toxicology studies in rats have established a NOAEL of 30 mg/kg/day for copper naphthenates (Table 2) (USEPA, 2018). Applying standard uncertainty factors of 10 for animal to human extrapolation and 10 for human variability, an equivalent to a HGV (such as a MRL or RfD) of 0.3 mg/kg/day is derived (Pohl and Abadin, 1995). Given this 0.3 mg/kg/day dose, the expected dose of copper is 0.034–0.040 mg/kg/day based on the percentage composition calculation above. The acute and intermediate oral MRLs of copper were 0.01 mg/kg/day (ATSDR, 2004a), so copper is slightly favored to be the driver of observed toxicity in this metal naphthenate as its dose will be 3-fold higher than that of copper alone. Zinc naphthenates are an alternative example considered here, where a developmental toxicology study in rats identified a NOAEL of 118 mg/kg/day (USEPA, 2007). By applying the same uncertainty factors as before, an equivalent HGV of 1.2 mg/kg/day is derived. Given this dose, the zinc cation dose would be 0.14–0.16 mg/kg/day based on percentage composition, but the intermediate oral MRL of zinc is twice this value at 0.3 mg/kg/day (ATSDR, 2005). Therefore, organic naphthenates may be responsible for the toxicological effects in the zinc formulation. Since there is also an MRL established for cobalt (0.01 mg/kg/day) (ATSDR, 2004b), equivalent HGVs can be derived for its corresponding naphthenates. It can also be determined if the toxicology is metal- or naphthenate-driven when copper and zinc naphthenate toxicology studies are used to determine the dose boundaries.

## 4 Discussion

Naphthenic acids are complex petroleum derivatives used in industrial and commercial products, but they are relatively uncharacterized in terms of their biological and toxicological properties. Although these chemicals are not considered to be undiscovered public health threats, they are one of many possible examples of UVCBs with high production and distribution in a society with numerous possible occupational and consumer exposure scenarios. The large data gaps associated with UVCBs are mostly responsible for the inability to provide health guidance. This is exemplified by several recent environmental exposures involving UVCBs that were investigated by the ATSDR, one of which was copper naphthenates exposure to dairy goats (ATSDR, 2021). Although the ATSDR was able to provide technical guidance for the goats to recover and be released from quarantine, handling this UVCB was a challenge as the normal tools that are rapidly employed for predictive assessments of discrete chemicals were generally inapplicable. We anticipate more environmental exposures involving UVCBs in the future and would therefore like to develop methodologies for their rapid assessments.

There are different frameworks and tools available to evaluate the chemical risks posed by UVCBs (European Chemicals Agency, 2017; House et al., 2021; Lai et al., 2022; Salvito et al., 2020). However, these generally require certain amounts of experimental toxicological data. For UVCBs lacking prior toxicological tests, there are large data gaps in the ability to provide health guidance even if these UVCBs are known to be relatively benign. This is where the application of cheminformatics tools that were primarily developed for pharmaceutical research can be utilized. For UVCBs like naphthenic acids, the tools developed to explore chemical analogs through combinatorial chemistry can be repurposed to develop virtual chemical libraries. The “enumeration tool” in Maestro (Schrödinger LLC, 2020b) is one such example, although other similar software choices are available in open-source packages, such as RDKit (https://www.rdkit.org/docs/RDKit_Book.html#chemical-reaction-handling). This approach is suitable for chemicals described by their core features and R-groups attached to the cores. Naphthenic acids can be deconstructed such that they have a center ring structure with an attached alkyl chain and alkyl acids. Thousands of UVCB constituents can be generated rapidly through this method, and modern cheminformatics database software can be used to easily manage virtual libraries of such sizes. It is possible that chemical entities created thus may have low molar fractions in the UVCBs or even be non-existent, but these could be filtered at later stages or allowed to remain in the virtual library if analytical evaluations of the UVCBs cannot rule out their presence in the UVCB. One method to check the compatibility of the virtual library with an actual UVCB is to compare the molecular weight distributions. In the case of crude naphthenic acids, the molecular weight distribution in the virtual library closely matched the experimental range of 240–330 g/mol. The virtual library contained a few components with lower and higher molecular weights, which could be an artifact of each component contributing equally to the virtual distribution compared to the actual UVCB, having varying mole fractions of each component depending on the source materials and production conditions.

With the availability of a virtual library of UVCB structures, it is possible to apply various structure-based tools and models to all the chemicals in the library. A first logical step in this case would be to check for available experimental information on the chemicals in the library. The EPA CompTox Chemicals dashboard is a convenient resource for searching for such matches. This can be accomplished through generating InChI keys for the structures and performing a batch search on the website, resulting in 14 matches. Although these 14 entries were identified, no experimental data of any kind (bioactivity, physicochemical properties, etc.) were available, highlighting the toxicology knowledge gaps for this specific chemical space. This precluded the direct use of *in vitro* to *in vivo* extrapolation (IVIVE) or designation of chemicals to biological mechanisms for better characterization of substances. It was possible to assess these 14 compounds using the GenRA web application and derive the read-across predictions. The mean POD values for the 14 matches were between 240 and 2,189 mg/kg/day. The lowest POD (most toxic) observed was 1.7 mg/kg/day for chronic cardiotoxicity, while the highest POD (least potent) was 4,531 mg/kg/day for chronic hematotoxicity. Although this is a limited toxicological view of naphthenic acids due to the lack of suitable matches in the database and low similarity of read-across surrogates, we obtain a possible range of expected toxicity values.

Although read-across could be performed only for a fraction of the library, established QSAR models can easily be implemented on thousands of naphthenic acid structures. These *in silico* tools that utilize computer-based modeling and simulation techniques have emerged as valuable resources for addressing the toxicological data gaps. Table 1 details all the cheminformatics tools and resources that have been used for predictive risk assessments of UVCBs along with some open-source alternatives to the commercial tools. These models and tools can be applied to the thousands of molecules in the virtual library, and the results can be interpreted for not only individual components but also entire mixtures. Consistent with the limited available experimental physical property values, such as complete solubility in organic solvents and limited solubility in water (<50 mg/L), the properties predicted by QikProp suggest high lipophilicities and low solubilities for most naphthenates (Figure 6) (Brient et al., 2000). OPERA 2.9 models can now be used to estimate ADME properties, such as hepatic intrinsic clearance and unbound plasma fraction; the results also provide a picture of the pharmacokinetic profile of the mixture, with relatively high expected plasma protein binding, consistent with the alkyl acids bound to albumin (Smith et al., 2019). Rapid intrinsic clearance in the liver is not predicted for any of the naphthenic acids; combined with protein binding, this suggests that the systemic clearance may be moderate to slow compared to more functionalized environmental chemicals.

Established QSAR models are also helpful for direct prediction of the toxicological endpoints and therefore provide a means of *in silico* toxicological screening. Systematic acute oral toxicity is an essential metric in evaluating the safety of a chemical and is used by many regulatory agencies. It is commonly measured experimentally through the median lethal oral dose (LD_50_) in rats. OPERA 2.9 now incorporates consensus models for predicting rat oral LD_50_ point estimates and hazard categories following a global collaboration effort in 2019. The predictions of this tool suggest low toxicity for all naphthenic acids, with LD_50_ point estimates between 2,000 and 9,000 mg/kg. Using published repeat-dose oral toxicity models, the chronic PODs in rats were predicted to be between 25 and 50 mg/kg/day, whereas the rat developmental PODs were predicted to be between 79 and 95 mg/kg/day. This latter prediction is concordant with reported rat developmental toxicity data for copper naphthenic acids (30 mg/kg/day NOAEL and 100 mg/kg/day LOAEL) (USEPA, 2018). Despite the experimental values being the results of complex mixtures dosed to rodents and predictions being the results for the entire virtual library, the predicted ranges nearly encompass both the experimental toxicological results. This indicates that the alkyl acid chemical space covered by the naphthenic acids may be well predicted in the repeated-dose models. However, based on the composition breakdown in Table 2, it is possible that the adverse effects are related more to the dose of copper than naphthenates, which would be consistent with copper being the active agent that provides fungicidal properties.

The QSAR predictions could not be directly compared with acute or repeat-dose toxicity for individual naphthenic acids owing to the lack of data. However, naphthenic acids have been tested as UVCBs in rat oral studies of both acute and repeat-dose toxicities (Sigma-Aldrich, 2022; Rogers et al., 2002). The experimental rat LD_50_ of 3,000 mg/kg for naphthenic acids was consistent with the predicted range of 2,000–9,000 mg/kg (Sigma-Aldrich, 2022). Rogers et al. (2002) observed adverse effects of naphthenic acids at 300 mg/kg in rats administered a single dose and 60 mg/kg/day in rats dosed for 90 days; these results are also consistent with the acute and repeat-dose toxicity predictions for the naphthenate portion, which was a rat oral LD_50_ > 300 mg/kg and a chronic POD between 25 and 50 mg/kg/day. Although this experimental toxicology approach is limited, it is concordant with the predictions and suggests that the overall methodology is appropriate for this UVCB.

Based on a rat developmental study for zinc naphthenates, which identified a NOAEL of 118 mg/kg/day (USEPA, 2007), it is deduced that zinc naphthenates have an equivalent to a HGV, such as an MRL or RfD, at 1.2 mg/kg/day (Table 2). At this level, the dose of zinc will be 4-fold below its intermediate oral MRL (ATSDR, 2005). Since metal cations and organic naphthenates are unlikely to have the same toxicological mechanisms, this indicates that naphthenates are the primary agents for the adverse effects over zinc and that an MRL-like value for this effect would be approximately 1.1 mg/kg/day. The naphthenic acid MRL for cobalt naphthenates would yield a dose of 0.12–0.14 mg/kg/day; this is more than 4-fold higher than cobalt’s oral MRL of 0.01 mg/kg/day (ATSDR, 2004b), so cobalt would likely drive the toxicology over the naphthenate components. The risk assessment should then focus on cobalt exposure and its health effects at doses near the cobalt MRL, although the effects from naphthenic acids should also be considered at higher doses. Interestingly, the average predicted rat developmental POD for naphthenic acids was 86 mg/kg/day, which is within 20% of the experimental result for the naphthenate portion of zinc naphthenates; this indicates that even without experimental information, a reasonable provisional assessment for human health guidance could be derived using predictions from the virtual chemical library. Based on the experimental results for zinc naphthenate, the naphthenic acid components appear to have similar toxicities to each other, in agreement with the *in silico* predictions. This low variability in predicted toxicity between the naphthenic acid components—4.5-fold for the acute and 2-fold for the repeat-dose predictions—is an interesting result (Figure 7). This could be an artifact from the “coarseness” of the global models for toxicity predictions trained on diverse datasets of environmental chemicals. Alternatively, it may simply be due to the similarities among the naphthenic acid components in the virtual library. UVCBs with more diverse structures would be expected to have greater variability in the predicted toxicities. Many UVCBs, however, have very similar structural components and would likewise have narrow variability in the toxicity predictions. Regardless, the agreement between the predictions and experimental results for this class of chemicals suggests high confidence in the models used herein.

There are several limitations to this methodology as applicable to metal naphthenates. The first limitation is that the percentage compositions of the organic components are not known, so concentration addition or toxic equivalent concentration methods cannot be applied. Most UVCBs lack the analytical characterization and data for these approaches (Lai et al., 2022). One possible method to overcome this limitation would be to assess the UVCB based on 100% composition of the most toxic component, although this would necessarily be very conservative. Weighting schemes are possible if there is analytical information on the fractions, such as aromatic or aliphatic components. Another major limitation is the assumption that there are no synergistic effects of the organic components. There were no obvious biological mechanisms by which these components would be synergistic, but this assumption should be noted. A final limitation is the requirement that the organic components of the UVCBs are within the applicability domains of the predictive models. More complex organics may not lie within these domains, and other types of substances such as polymers, biologics, and complex organometallics are known to be outside the applicability domains of the models used in these predictions. Most QSAR models, including the ones used here, are built on the basis of chemical descriptors or fingerprints generated for small-molecule organic compounds in the training sets. These models may also be sensitive to the exact compounds in the training set, errors in the data, or machine learning algorithms applied and their precise tuning. Thus, the QSAR model chosen in this application should be viewed as an ongoing evolution of the technique and not fixed for these specific models.

Even with these limitations, there are many advantages to the proposed methods of chemical elucidation of UVCBs (cheminformatics tools and databases). These methods enable the grouping of components based on structural similarities, predicted fates and transport, and predicted biological and toxicological properties. They could be coupled with other new methodologies to identify the main drivers of effects for a UVCB with constituent or fractional profiling (House et al., 2021; Lai et al., 2022). Once specific fractions or constituents of concern are identified, further toxicological assessment may be performed for a much reduced number of chemical entities. When the PODs are determined for these entities, a risk assessment could be inferred for the entire UVCB based on whether the entities have the same modes of action (concentration addition) or independent action models of toxicity (Lai et al., 2022).

Many applications can be envisioned for the proposed methodology. If an environmental spill involving a UVCB requires a chemical risk assessment, this approach can be applied immediately if the UVCB matches one of the first three structural description categories defined by Lai et al. (2022): well-defined individual structures, descriptive representative structures, and recognized external identifiers with some standardized descriptions. The fourth structural category of simple text-based descriptions would require additional analytical determination of the UVCB components before application. For all categories, an understanding of the reagents and chemical reactions producing the UVCB would be very helpful in defining the enumeration steps to produce the library of compounds in a UVCB. If the UVCB is sufficiently characterized, then its compound library can be generated through enumeration using either Maestro or RDkit (Table 1). The tools described above for read-across, ADME, acute toxicity, and repeat-dose toxicity predictions can then be applied (Table 1; Figure 2). Using these results, a provisional risk assessment could be made and applied to the situation. Non-emergency scenarios that may possibly benefit from this methodology are hazardous waste sites containing UVCBs that require assessments, residential or public areas that are near UVCB sources (fenceline concentrations), and food or water sources that may become contaminated by UVCBs. Without a suitable methodology for evaluating UVCBs, these risk assessments are severely limited by the lack of toxicological information for a whole-substance UVCB and the sheer number of UVCB components. Although exciting new approaches for risk assessments like the EPA Transcriptomic Assessment Product (ETAP) are gaining traction and could be applied to UVCBs, these methods still require animal testing and may take months for completion (Brennan et al., 2024). Thus, the proposed method could provide more rapid responses while other studies are underway.

## 5 Conclusion

The results from the present study suggest that the predictions of a virtual chemical library can be helpful tools for assessment of UVCBs with limited experimental toxicological information. One of the key steps in this work is building a virtual library encompassing as many of the components of a UVCB as possible, which would require analysis and chemical expertise. For metal naphthenates as UVCBs, these predictions can be used with the results of experimental toxicological studies for greater insights into the dual effects of dissociated metals and naphthenates. Such insights are especially possible when the corresponding metals already have HGVs, allowing comparisons with the predicted toxicology of the naphthenate portion. This implies that metals are often the key determining factors of exposure in the case of toxic metals, while the naphthenate portion may be the more critical component when present with less toxic metals. This approach can be applied to other appropriate UVCBs, such as bromo-chloro alkene flame retardants, chlorinated paraffins, and other petroleum derivatives (Chibwe et al., 2019), thereby advancing their risk assessments with predictive toxicity tools and *in silico* screening. A similar concept may be applied to *in vitro* whole-substance screening, which could support mechanistic interpretations of the computational data. These predictions should be limited to provisional guidance and should not replace experimental assessments. Further validations with various UVCBs is necessary to demonstrate the broader applicability of the proposed methodology.

## Data Availability

The original contributions presented in the study are included in the article/Supplementary Material; further inquiries can be directed to the corresponding author.
